# Development and Evaluation of a 3D-Printed Adult Proximal Tibia Model for Simulation Training in Intraosseous Access

**DOI:** 10.7759/cureus.12180

**Published:** 2020-12-20

**Authors:** Reniel Engelbrecht, Chris Patey, Adam Dubrowski, Paul Norman

**Affiliations:** 1 Medicine, Memorial University of Newfoundland, St. John's, CAN; 2 Emergency Medicine, Carbonear General Hospital, Carbonear, CAN; 3 Health Sciences, Ontario Tech University, Oshawa, CAN

**Keywords:** emergency medicine and trauma, simulation in medical education, three-dimensional (3d) printing, task-trainer, intraosseous

## Abstract

Intraosseous infusion remains an underutilized technique for obtaining vascular access in adults, despite its potentially life-saving benefits in trauma patients and those presenting to the emergency department. There is a scarcity of cost-effective, anatomically correct trainers to improve physician confidence and competency in this skill. The purpose of this report is to describe the development and evaluation of a three-dimensional (3D) printed Adult Proximal Intraosseous (IO) Tibia task trainer for simulation-based medical education. The proposed trainer was designed by combining open-source models of a human skeleton and a lower leg surface scan in Blender (Blender Foundation - www.blender.org) and manipulating them further using a JavaScript program. Polylactic acid was used to simulate bone while cured silicone moulds were used to replicate skin and soft tissue. Two trainers were produced and tested by 15 rural family medicine residents, six rural emergency medicine physicians, and six registered nurses. Participants evaluated the realism of the trainer and its efficacy as a training tool through a structured survey.

The trainer received overall positive feedback from all participants, and most participants felt that no improvements were required to use the trainer for medical education. Notable suggestions for improvement included adding an infusion component, increasing the size of the tibial tubercle for better landmarking, and creating a variety of sizes for different patient body types. Residents and emergency medicine physicians practising in rural Newfoundland and Labrador found the 3D-printed trainer to be a practical tool for practising intraosseous technique. The outcome of this report supports the use of this cost-effective trainer for simulation-based medical education.

## Introduction

Simulation-based medical education (SBME) is a rapidly growing field that employs realistic simulators to allow physicians and medical students to practice clinical procedures without causing unnecessary patient harm [1]. The introduction of three-dimensional (3D) printing into medical education presents a low-cost alternative to expensive simulators for practising procedural skills. Anatomically correct models can be created from 3D reconstructions of MRI and CT scans or open-source resources online [2], allowing for accurate landmarking and positioning using real anatomical features. Specialities such as Emergency Medicine, especially in rural communities, can benefit from SBME by allowing learners and professionals to learn and practice rare but potentially life-saving skills in a controlled and safe environment. One such high acuity, low occurrence (HALO) skill is intraosseous (IO) infusion.

By infusing fluid directly into the non-collapsible bone marrow cavity, from where it is rapidly absorbed into the circulation, IO access provides an alternative route to vascular circulation when peripheral intravenous (IV) access is compromised. This is a common issue in patients with severe dehydration, small veins, or peripheral vascular collapse [3]. Consequently, IO is primarily used in the Emergency Department (ED), where rapid vascular access is of critical importance for patients presenting with shock, major trauma, cardiac arrest, and other conditions requiring emergent fluid or drug administration. IO access is superior in both success rate and access time than other alternative routes, such as central venous catheterization (CVC) [4,5]. As such, both the 2010 American Heart Association Guidelines for Advanced Cardiovascular Life Support (ACLS) and European Resuscitation Council Guidelines recommend using IO access for drug administration when IV access is difficult or impossible [5,6]. Despite these recommendations, it is often an underutilized procedure in the adult population, used only as a fourth option after multiple failed attempts at obtaining peripheral IV access and CVC [7]. When Danish researchers investigated reasons for lack of IO use in situations where it was medically necessary, in a cohort of 761 physicians, nurses, and emergency responders, they found that lack of training was the second most common response after lack of equipment [8].

Furthermore, a recent survey of 644 fourth-year US medical students found that 95% had never attempted an IO insertion, and 92% either felt utterly uncomfortable with the procedure or would seek supervision to complete it [9]. A study from France suggested the use of the simulated practice in improving performance capabilities when experienced physicians who practised IO insertion on artificial bones and mannequins obtained significantly higher scores (mean score: 19.13/20) on a validated performance assessment scale compared to novice trainees (mean score: 11.06/20) [10]. Other studies have also supported the early introduction of intraosseous insertions in medical training using cadavers or manikins to increase competent use [11].

The availability of natural, cost-effective materials for practising IO infusion is limited. Current tools include low-fidelity models provided in practice kits by IO drill manufacturers, such as the BIG (PerSys Medical) training kit, consisting of a practice needle and foam block [12], and high-fidelity trainers such as the “Intraosseous Leg Trainer” by Laerdal® (Laerdal, Stavanger, Norway), for which a 10-pack of disposable tibial segments cost 180 USD [13]. Turkey and chicken bones also serve as cheap and accessible practice tools, but they lack features such as skin and bony landmarks that add realism to the experience. 3D printing provides the means to create a cost-effective, anatomically correct trainer for practising this HALO procedure where existing high-fidelity trainers may be difficult to access.

This technical report aims to describe the development and evaluation of the first iteration of a novel 3D-printed Adult Proximal Tibia IO Trainer. The proximal tibia has been shown to result in the highest success rate, is superficial, and does not interfere with ongoing cardiopulmonary resuscitation (CPR) [11]. This study aimed to obtain feedback from content experts with experience in IO infusions to evaluate the trainer’s realism and its efficacy as a potential training tool to optimize its design for use in medical education.

## Technical report

**Figure 1:**
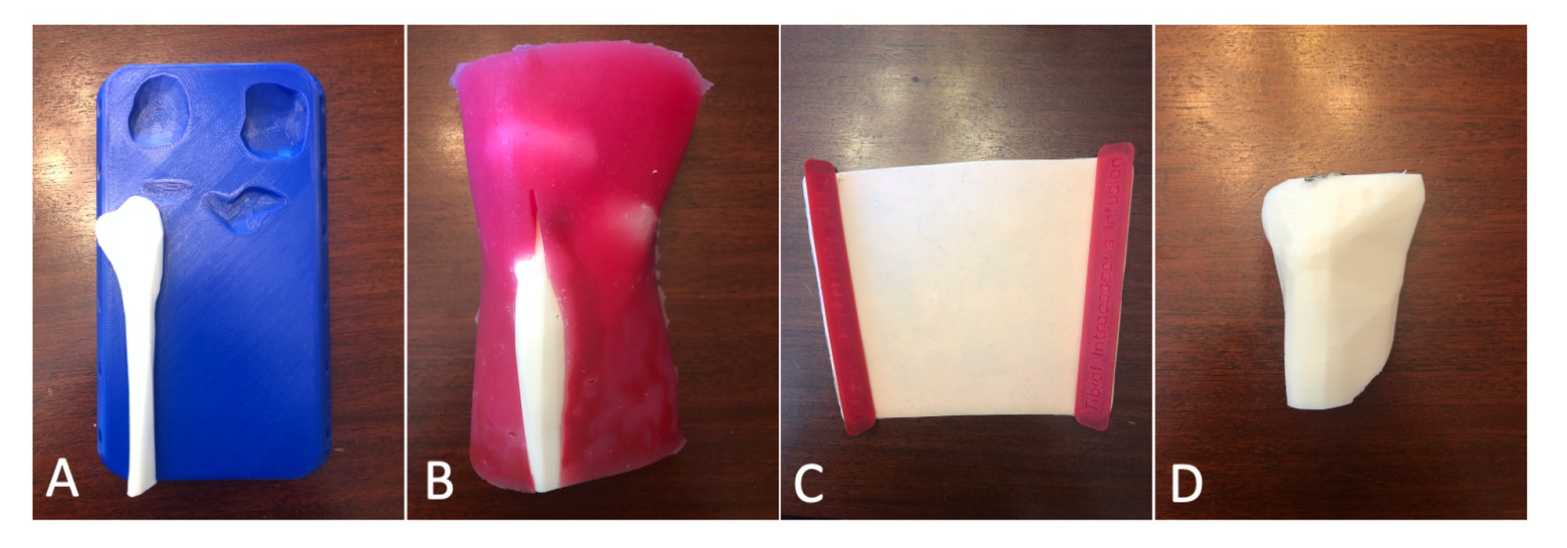
Components of the Adult Proximal Tibia IO Trainer A) Base; B) bone and muscle tissue; C) skin attachment; D) replaceable tibial cartridge

The novel Adult Proximal Tibia IO Trainer is composed of four main parts (Figure 1): a base unit, a bone and muscle tissue unit, a replaceable tibial cartridge, and a replaceable skin attachment. All 3D-printed parts were printed in polylactic acid (PLA) or thermoplastic elastomer (TPE), using a Prusa i3 MK3S or an Ultimaker 2+ 3D printer. All silicone parts were made using a mix of Ecoflex™ 00-30 (1:1 A:B ratio, with a small amount of pigment).

The design was derived from two anatomical models, a full skeleton and a lower leg surface scan, shared online through a Creative Commons license (Creative Commons, Mountain View, CA, USA). The two models were carefully aligned in Blender (Blender Foundation - www.blender.org). A Boolean intersection, an operation that combines two or more meshes and preserves only the parts of each mesh that overlap, was applied to the skeleton and a simple cuboid to extract the portions of the four bones articulating at the knee (femur, tibia, fibula, and patella) required for the model. A similar, more simple cuboid was used to isolate the soft tissue from the leg surface scan. The meshes were cleaned up in Meshmixer (Autodesk Inc., San Rafael, CA, USA), and the four bones were split into separate stereolithography (STL) files. A JavaScript program was written, utilizing the open-source library OpenJSCAD, to manipulate the meshes. This code-based approach allowed for rapid modifications to the bounding cuboid.

**Figure 2:**
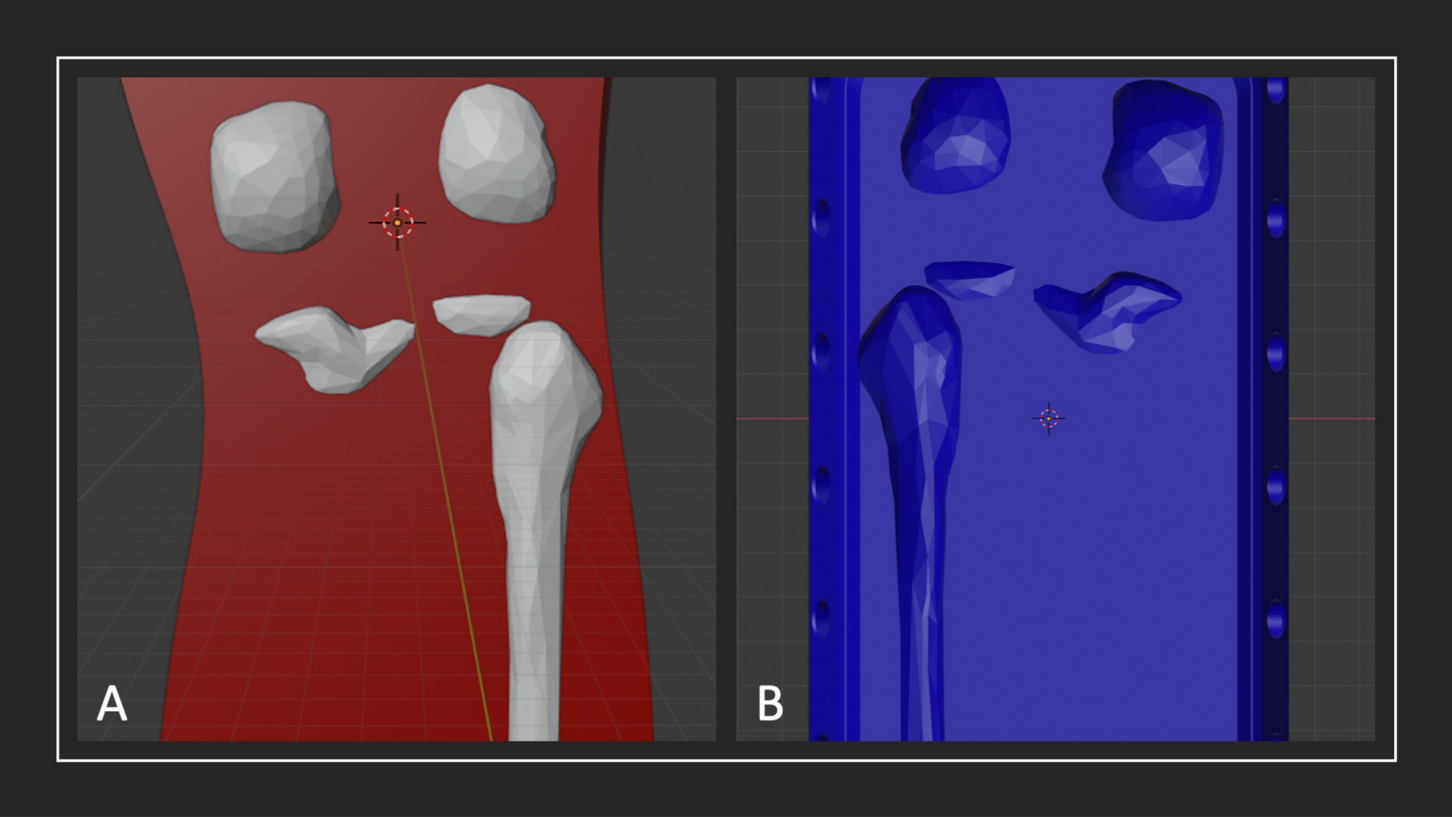
Screenshots of model rendering in Meshmixer A) Rendering of cured silicone muscle mould with bones; B) rendering of base unit with recessed shapes from boolean subtraction of bones

Cured silicone was shaped into a mould that left four voids for the plastic bones to fit into (Figure 2A). Boolean subtraction was used to create the base unit with recessed shapes for the protruding bones, as well as holes for the pegs of the fasteners used to attach the skin (Figure 2B). The fastener and skin mould were generated in OpenJSCAD. The fastener was printed in TPE to provide flexibility and durability. The silicone skin mould was designed as a trapezoidal shape with twelve holes along the side edges (six per side) to attach to the fastener and subsequently to the base, providing realistic skin tension and geometry.

In total, 1132g of PLA, 19g of TPE, and 835g of silicone were used to produce each model, including moulds and the skin tray. The longest print time of any component in this trainer was 17 hours and 49 minutes for the muscle mould, and the total material cost was roughly 65 CAD per trainer. Rather than replacing the entire trainer, a tibial cartridge was designed on the tibia’s anteromedial surface. This cartridge uses only 23g of PLA and costs 0.70 CAD of material per unit.

Context

Feedback on the Adult Proximal Tibia IO Trainer was collected in two separate data collection sessions. The first session was the 2019 Academic & Wellness Resident Workshop held at the Bay Roberts Hotel and Tilt Room on November 23, 2019. The workshop was held for family medicine (FM) residents practising in rural Newfoundland and Labrador (NL). The workshop included a procedural and trauma simulation session where residents were offered the opportunity to practice specialized procedures required by rural ED physicians (FM is a standard route to practising rural emergency medicine in NL). Two Adult Proximal Tibia IO Trainers were set up as a simulation station for this session. The second data collection session took place at the Carbonear General Hospital ED in Carbonear, NL. ED physicians and registered nurses (RN) were given the opportunity to test the trainer. In total, 15 FM residents (twelve PGY1 and three PGY2), six ED physicians, six RNs, and one unspecified practitioner participated in the study.

Inputs

**Figure 3:**
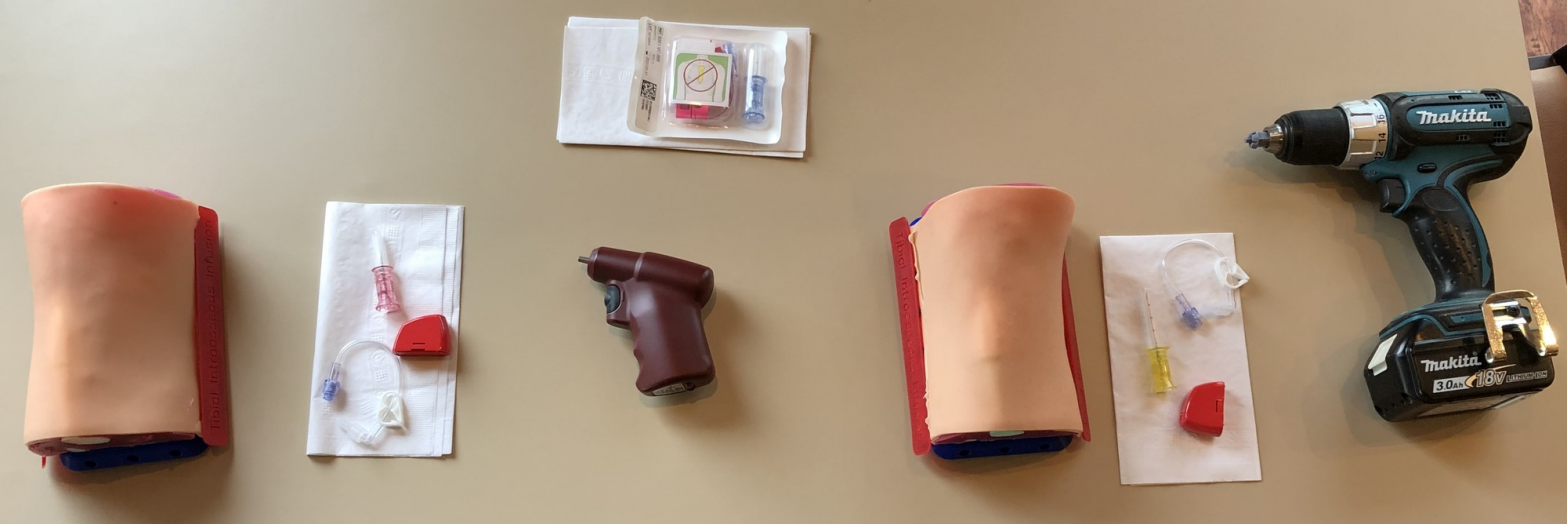
Equipment setup showing two Adult Proximal Tibia IO Trainers, EZ-IO power driver and needle sets, and cordless drill

The equipment provided to participants included: Arrow® EZ-IO® Power Driver (Teleflex Medical Research Triangle Park, NC, USA), EZ-IO® Needle Set, Makita 18V lithium-ion cordless drill with custom 3D-printed drill bit made to fit EZ-IO® needle, and the Adult Proximal Tibia IO Trainer (Figure 3). The participants were given an instructional demonstration by the primary investigator on appropriate landmarking, needle insertion, and attaching the catheter hub.

Participants were also provided with a 15-question product evaluation survey to complete after using the trainer, consisting of a combination of Likert-type and open-ended questions (Table 1).

**Table 1:** Questions of the product evaluation survey (not shown: Section Three consisting of an open-ended comment box for suggestions and feedback). *Section Two was completed only by those participants with prior experience using pre-existing trainers.

Adult Proximal Tibia IO Task Trainer					
Q1: Realism Evaluation					
For the following questions: 1 = Not at all realistic; 3 = Adequate realism, but could be improved; 5 = Highly realistic, no improvements required					
Physical Attributes	1	2	3	4	5
Anatomical Structure					
Colour					
Shape					
Texture					
Size					
Material					
Overall appearance					
Realism of Experience	1	2	3	4	5
Landmarking insertion point					
Inserting the Needle					
Q2: Have you practiced or performed these procedural skills before? (Yes or No):					
# of times procedure performed:					
Setting (i.e. emergency room/operating room):					
For the following questions: 1 = Not at all effective; 3 = Effective; 5 = Very effective					
	1	2	3	4	5
Q3: In your opinion, how effective is this new Adult IO Trainer in increasing trainees’ skills-based competency in IO insertion?					
Q4: In your opinion, how effective is this new Adult IO Trainer in increasing trainees’ confidence to perform IO insertion?					
	Yes			No	
Q5: Did the new Adult IO Trainer help you to understand something you didn’t previously understand about IO insertion?					
Q6: Would you use this new Adult IO Trainer to assist with your ongoing training and/ or education?					
Q7: Would you recommend the use of this new Adult IO Trainer to assist with the training and education of other professionals and students?					
For the following question: 1 = Not at all relevant; 3 = Relevant; 5 = Very relevant					
	1	2	3	4	5
Q8: Please rate the value of the Adult IO Trainer as a training tool.					
Q9: Global Rating – Please check the one statement below with which you most agree:					
The simulator requires extensive improvements before it can be considered for training					
The simulator requires minor improvements before it can be considered for training					
The simulator requires no improvements and can be used for training					
Section Two: Comparison of Adult IO Trainer to the pre-existing IO task trainers*					
Q10: Which pre-existing IO Task Trainers have you used to practice IO insertion? (Please briefly describe):					
For the following questions: 1 = Far below standards; 3 = Meets standards; 5 = Far above standards					
	1	2	3	4	5
Q11: In your opinion, how effective is the new Adult IO Trainer in increasing trainees’ skills-based competency in IO insertion compared to the pre-existing IO Task Trainers?					
Q12: In your opinion, how effective is this new Adult IO Trainer in increasing trainees’ confidence to perform IO insertion compared to the pre-existing IO Task Trainers?					
Q13: How does the “realism” of the new Adult IO Trainer compare to the pre-existing IO Task Trainers?					
	Yes			No	
Q14: In your opinion, do you feel that the new Adult IO Trainer is a useful adjunct to the pre-existing IO Task Trainers?					
Q15: In your opinion, do you feel that the new Adult IO Trainer is a good replacement for the pre-existing IO Task Trainers?					

Process

**Figure 4:**
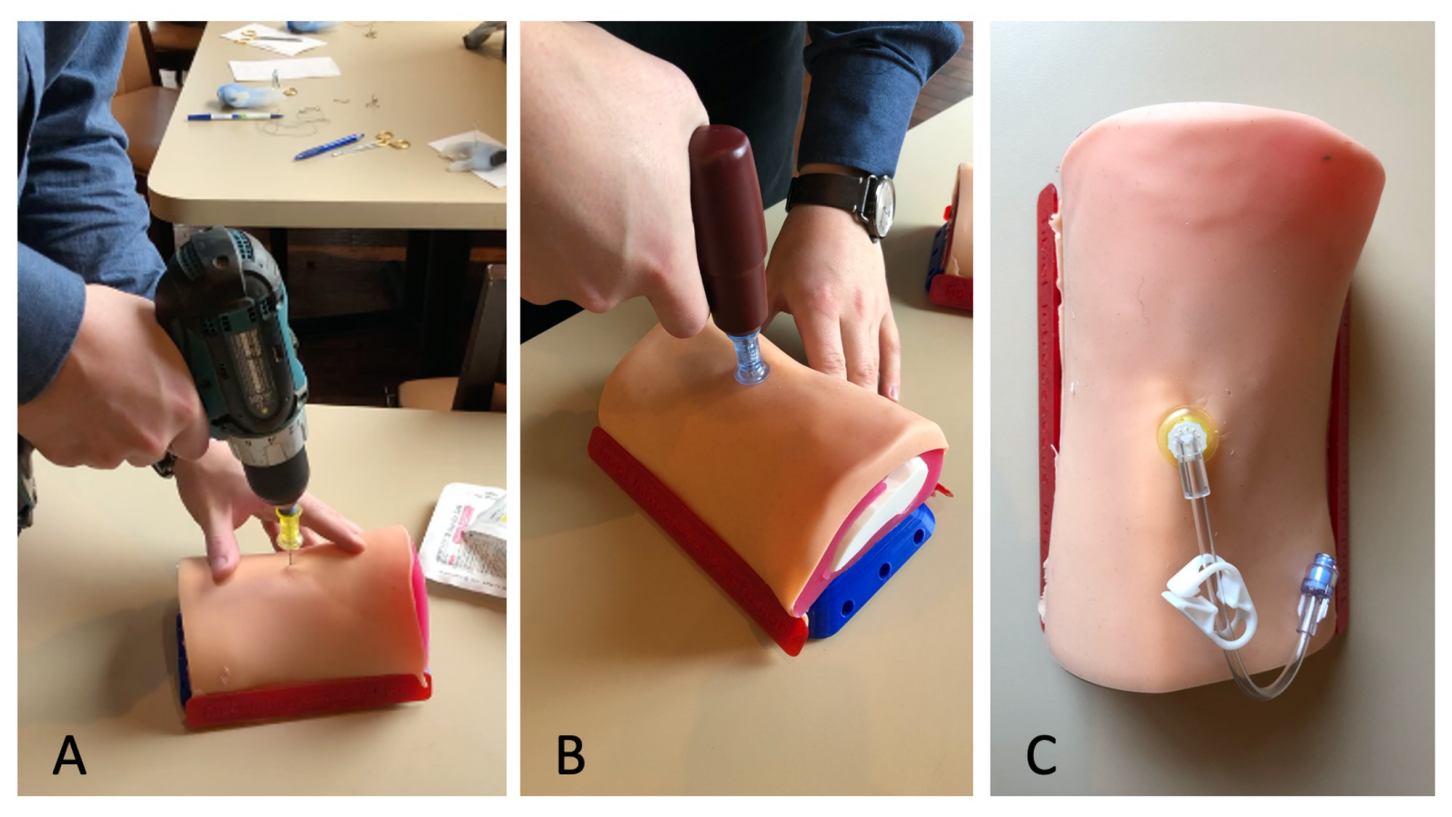
Testing of Adult Proximal Tibia IO Trainer A) Testing with cordless drill and custom drill-bit; B) testing with EZ-IO Power Driver; C) trainer following needle insertion

Following the instructional demonstration, the participants were asked to practice IO needle insertion on the model using both the Arrow® EZ-IO® Power Driver and the cordless drill (Figure 4). They were allowed to practice the procedure as many times as they wished during a five-minute period, after which they were asked to complete the survey. All responses to the product evaluation survey were analyzed using Microsoft® Excel for Mac (Microsoft, Version 16.36). As RNs are not trained to perform IO infusions, their data was only included to assess the trainer's physical attributes (i.e., Q1).

Products/Outcomes

**Figure 5:**
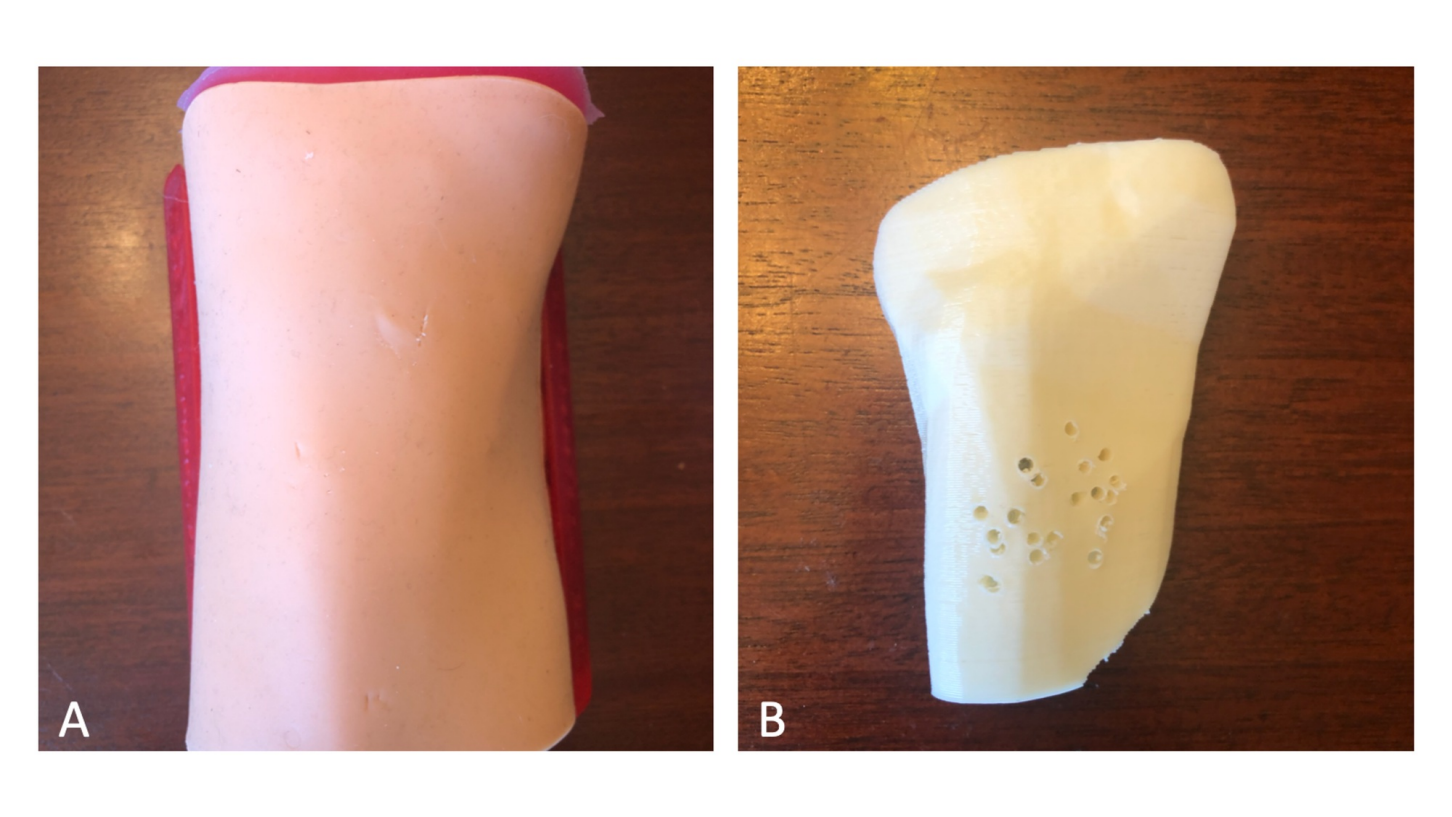
Photos showing wear of the Adult Proximal Tibia IO Trainer following data collection A) Skin attachment; B) tibial cartridge

Two Adult Proximal Tibia IO Trainers were fabricated. Despite numerous IO needle insertions by all participants, the disposable tibial cartridge and skin attachments showed little wear and did not require replacing throughout the entire duration of testing (Figure 5). Of the 28 medical professionals who participated in the study, 27 completed the survey. The results of the three sections of the survey are presented below.

Question one aimed to evaluate how accurately the trainer looked and felt like the real procedure, using nine components to describe its realism on a 5-point Likert-type scale (Table 2). The remainder of the survey focused on evaluating the trainer’s utility as a medical education tool. Only data from physicians and residents were included.

**Table 2:** Results of realism evaluation by ED physicians, FM residents, and RNs N = number of participants; SD= standard deviation; FM- family medicine; ED- Emergency Department; RN- registered nurses

Question on the product evaluation survey	Mean ± SD		
Physical Attributes	Physicians (N=6)	Residents (N=15)	Nurses (N=6)
Anatomical structure	4.7 ± 0.5	4.2 ± 0.8	5 ± 0
Colour	5 ± 0	4.3 ± 0.6	5 ± 0
Shape	4.8 ± 0.4	4.3 ± 0.6	5 ± 0
Texture	4.8 ± 0.4	4.2 ± 0.7	4.7 ± 0.8
Size	5 ± 0	4.3 ± 0.6	5 ± 0
Material	5 ± 0	4.3 ± 0.7	4.8 ± 0.4
Overall appearance	5 ± 0	4.3 ± 0.6	5 ± 0
Realism of Experience			
Landmarking insertion point	4 ± 0.6	4 ± 0.7	N/A
Inserting the needle	4.3 ± 0.8	4.3 ± 0.5	N/A
Likert Scale: 1 = Not at all realistic; 3 = Adequate realism, but could be improved; 5 = Highly realistic, no improvements required.			

Question two inquired about participants' previous experience with the IO procedure. All six ED physicians reported having performed IO insertions in the clinical setting. In contrast, eight of the 15 FM residents (53%) had previously practised the procedure, only one of which was in the clinical setting.

**Figure 6:**
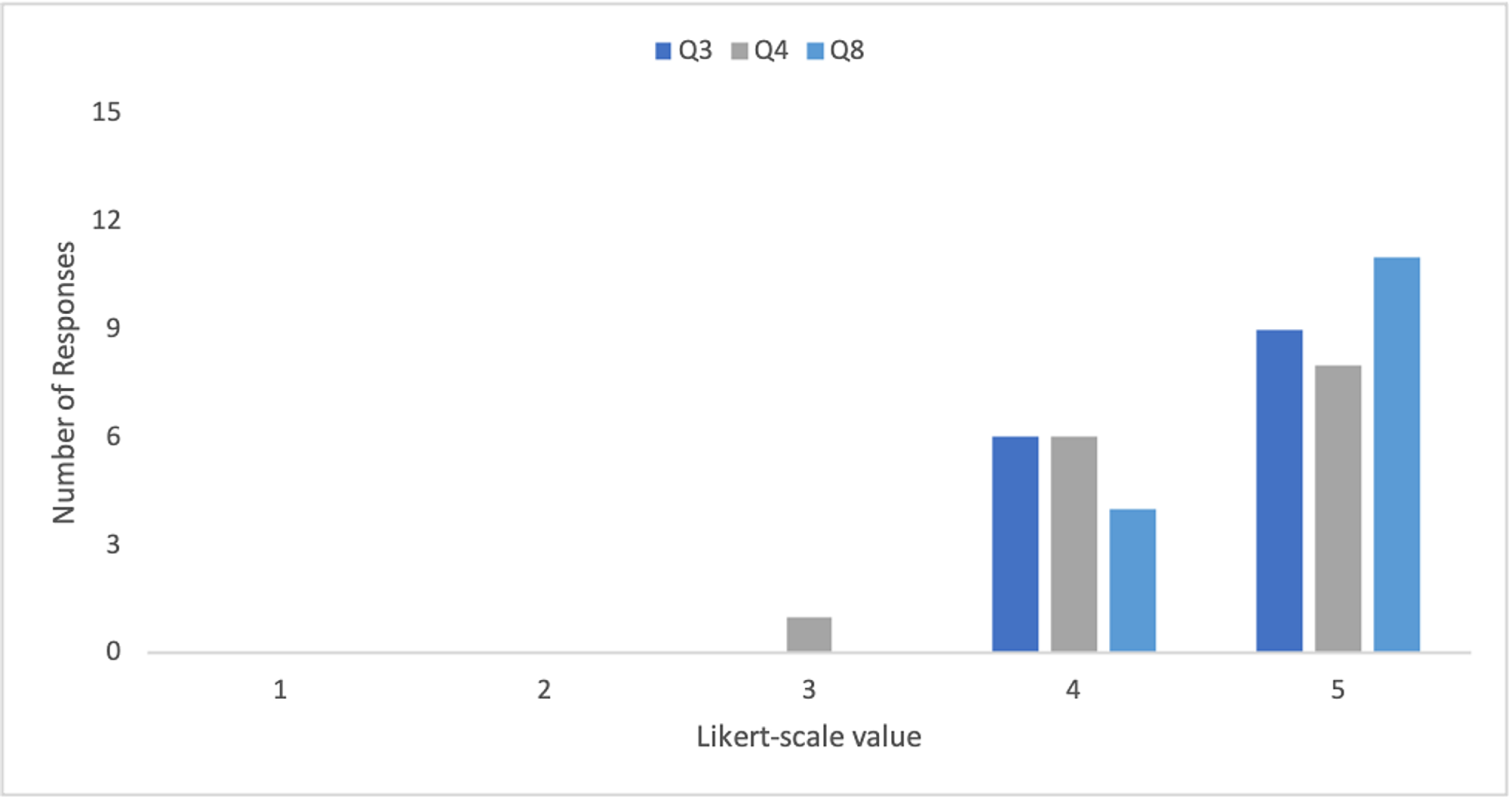
Frequency of FM resident responses to questions 3, 4, and 8 of the product evaluation survey (N=15) Q3: In your opinion, how effective is this new Adult IO Trainer in increasing trainees’ skills-based competency in IO insertion? Q4: In your opinion, how effective is this new Adult IO Trainer in increasing trainees’ confidence to perform IO insertion? Q8: Please rate the value of the Adult IO Trainer as a training tool. Likert scale: 1 = Not at all effective; 3 = Effective; 5 = Very effective. FM- family medicine

When asked to rate the trainer's efficacy and value as a potential training tool (Q3, Q4, & Q8), all six ED physicians considered the trainer to be very effective (i.e., rated 5 out of 5 on the Likert scale). The distribution of ratings by FM residents showed that the majority considered the trainer to be very effective (Figure 6).

**Figure 7:**
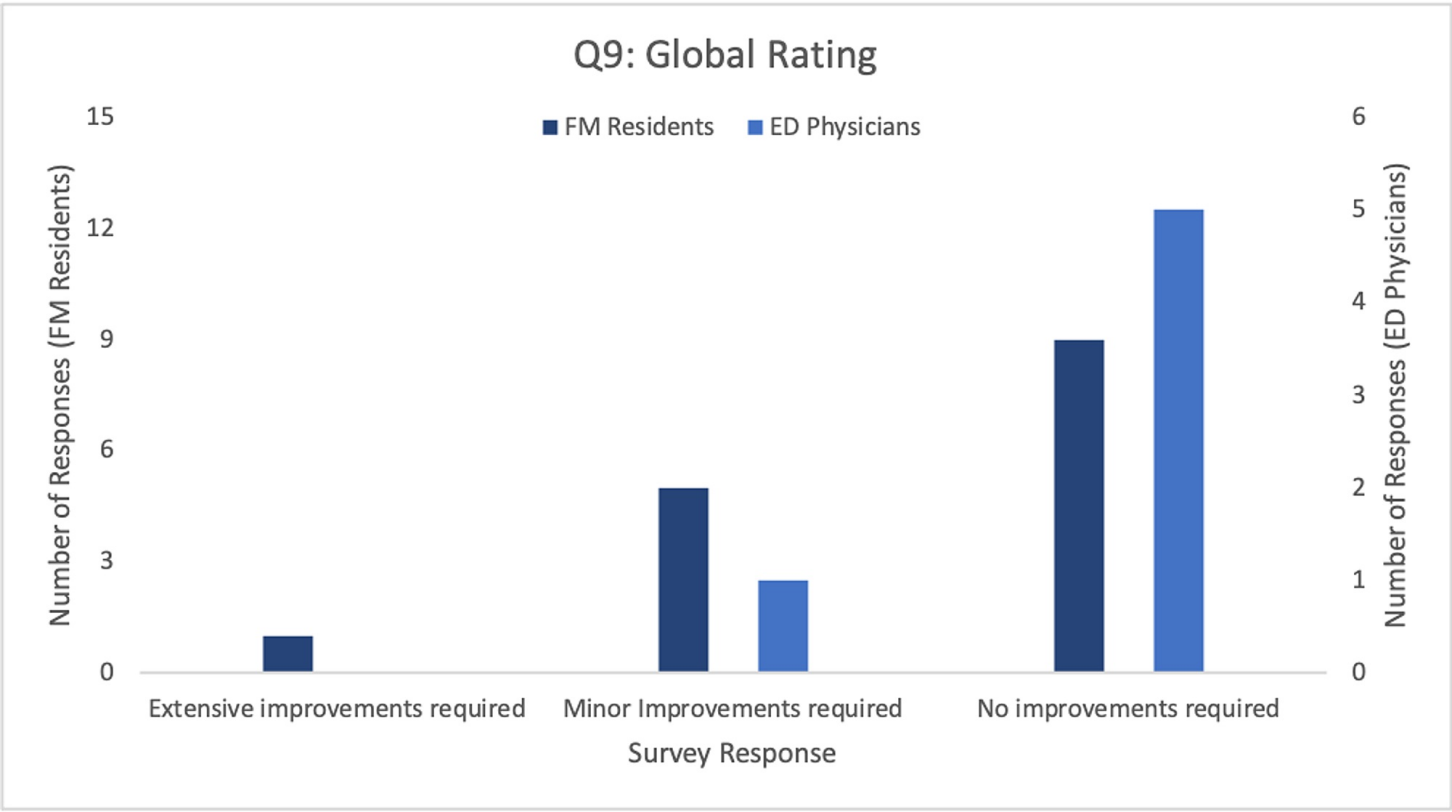
Frequency of responses by FM residents (N=15) and ED physicians (N=6) to Question 9 of the product evaluation survey FM- Family Medicine; ED- Emergency Department;

Questions five through seven asked if participants felt they had learned something about the procedure through using the trainer, and whether they would recommend it as a training tool. Three of the six ED physicians (50%) and 10 of the 15 FM residents (67%) reported that the current IO trainer helped them understand something they did not previously understand about IO insertion. All participants in both groups indicated that they would use the current trainer to assist their ongoing training and education, as well as recommend its use for other professionals and students. Question 9 aimed to determine the extent of improvement required before the trainer may be used as a training tool, and found that the majority of participants felt no improvements were required (Figure 7).

The second part of the survey was only completed by those with experience with pre-existing IO task trainers. Three ED physicians (50%) and six FM residents (40%) reported having used a pre-existing task trainer and completed this section. Practice materials included trauma simulation models, plastic bone models without overlying tissue, and higher fidelity IO trainers for pediatric patients. Questions 11 through 13 asked participants to compare the current trainer’s efficacy in increasing trainees’ competency and confidence in IO insertion and its realism compared to other trainers. Eight of the nine participants felt that the proposed trainer was above standards in all areas and is both a useful adjunct and a good replacement for pre-existing IO task trainers. The remaining participant felt that the proposed trainer met standards in these areas.

Participants were encouraged to provide suggestions and feedback in an open-ended comment box. Overall, the reception was positive, with responses stating that the trainer was “helpful,” “excellent,” and “very realistic.” Two residents commented that they appreciated the opportunity to practice a skill they “don’t practice often” and is “otherwise hard to practice.” Emergency medicine physicians suggested that it would be “helpful to have infusion component” and a “more prominent tibial tubercle for landmarking.” Notable suggestions from residents included: “difficult to landmark to get a sense of anatomy,” “better skin, it twists,” “add more realism … use the sterile technique”, and “due to our patient population it would be useful to have models that represent obese patients.”

## Discussion

The Adult Proximal Tibia IO Trainer received largely positive feedback and was considered a useful training tool by both FM residents and ED physicians. The trainer demonstrated excellent reusability, showing only slight wear of the disposable components. Some participants felt that the trainer could use improvements to enhance the realism of the experience. The lowest ratings for realism were seen in the “landmarking insertion point” component, a criticism supported by the comments above. Overall, the results show that the trainer is representative of the procedure to both those with ample experience in IO technique and those with limited exposure, but that it may require improvements to be a more effective training tool for less experienced learners. Based on the comments, recommendations for future iterations of the trainer include: improving anatomical accuracy to allow for better landmarking (i.e., palpation of the tibial tubercle); adjusting the silicone skin attachment so that it does not twist when the needle is inserted; adding the capability to attach an infusion line to flush and inject fluids; and improving the realism of the experience by using sterile technique and creating anatomical variations to simulate different body types.

Only nine of the 24 residents and physicians had used a pre-existing IO trainer, and all nine affirmed the 3D-printed trainer either met or exceeded the standards set by the pre-existing trainers. In comparison to existing trainers, ten tibial cartridge replacements of the 3D-printed trainer can be printed for 7.00 CAD, as opposed to buying them online for 180 USD/10-pack from Laerdal® [13], or 57 USD each from GTSimulators (GTSimulators, Florida, USA) [14]. In addition to its low cost, an advantage of this 3D-printed trainer is that it can be readily reproduced in rural centres with access to a 3D printer, such as the Carbonear Institute for Rural Research and Innovation by the Sea (CIRRIS), located in Carbonear, NL. This reproducibility makes it possible for rural medicine trainees to practice rare but potentially life-saving procedures, building confidence and competence in their skills before using them in practice. Furthermore, the model showed minimal wear, making it suitable for repeated use to maintain this skill over time.

The results of this study should be interpreted within the context of its limitations. A relatively small cohort of individuals, all practising within the same province, participated in this study. Additionally, approximately only half of the residents had previously practised the IO technique, and most of these had only done so in simulation. The lack of training in the resident group supports the findings by others that IO insertion is an undertrained technique among medical learners [9]. However, it also limits their ability to assess the realism of the trainer. While only a small cohort of ED physicians participated in the study, they possessed the experience of real content experts, and their feedback is most significant to the evaluation of this trainer. Their experience allows them to comment on the realism of the model, and to identify if any critical features necessary for effectively practising this procedure have been overlooked. Multiple studies have highlighted the need for increased training to improve utility and user competence in IO infusion [8-10,15].

Contrary to these findings, a survey distributed to trauma and emergency medicine physicians in Canadian, Australian, and New Zealand found that 98% of respondents had received training with IO devices, and 79% were comfortable administering an IO line at the proximal tibia [16]. The researchers concluded that there is no apparent lack of IO application in practice. However, the results are limited by potential non-responder bias, as the survey only achieved a 24% response rate. Additionally, there was no distinction between physicians practising rurally or at large urban centres, which could be a substantial factor when considering their exposure to the procedure.

## Conclusions

This report described the development and evaluation of a 3D-printed Adult Proximal Tibia IO trainer. The intent was to assess the realism of experience and obtain suggestions from content experts to improve the trainer and produce a useful teaching tool. Overall, the results indicated that both FM residents and ED physicians felt that the trainer would be a valuable and useful medical education training tool. Both groups made several suggestions to improve the realism of the trainer. After implementing these improvements and evaluating the trainer’s validity, it could be used to train medical learners and professionals in an otherwise infrequently used procedure, even those in rural locations, with the end goal of increasing the utilization of this procedure when indicated in practice.
